# MRI- and PET-Based Assessment of Radiological and Clinical Factors Associated With Cervical Cancer Response to External Beam Radiation Therapy

**DOI:** 10.7759/cureus.30645

**Published:** 2022-10-24

**Authors:** Arun G Paul, Steven Miller, Lance K Heilbrun, Daryn W Smith

**Affiliations:** 1 Radiation Oncology, University of Alabama at Birmingham, Birmingham, USA; 2 Oncology, Wayne State University School of Medicine, Detroit, USA; 3 Biostatistics and Bioinformatics Core, Karmanos Cancer Institute, Detroit, USA

**Keywords:** external beam radiation therapy, tumor regression, mri, radiation response, pet, fdg-suv, cervical cancer

## Abstract

Introduction

In the era of MRI-guided external beam radiation therapy (EBRT), complete radiological response (CR) is often seen in cervical cancer (CC) with 4-6 weeks of chemotherapy and EBRT. The clinical and radiological factors associated with this observation were investigated in this study.

Materials and methods

One hundred and twenty-four CC patients treated with concurrent chemotherapy, EBRT, and brachytherapy (BT) from January 2008 to July 2015 were retrospectively screened. Initial primary gross tumor volume (GTV_INITIAL_) was estimated after contouring on a planning CT scan registered with pre-EBRT PET and MRI. The maximum standardized uptake value (SUV) of GTV_INITIAL_ from each PET scan report was collected. Spearman's rank correlation coefficient (rho) values were calculated to assess the relationships among age, tumor size, and SUV. Tumor radiological response during EBRT prior to BT was calculated by contouring the final primary gross tumor volume (GTV_FINAL_) using MRI obtained prior to BT. CR rates during EBRT were estimated from GTV_INITIAL_ and GTV_FINAL _and compared by the level of various factors using Fisher’s exact test (two-sided).

Results

Forty-eight patients met the inclusion criteria of the study with a median age of 50 years. The median GTV_INITIAL_ was 82 cc. The median SUV was 14.9. A significant correlation was seen between SUV and GTV_INITIAL _with a larger tumor size associated with a higher SUV. CR rates were numerically higher for patients who were aged <50 years, or with >37.5 Gy radiation dose at or before the second MRI, or with GTV_INITIAL_ <100 cc, or with no nodes involved or with stages IB or IIA.

Conclusions

Our study identified higher CC primary tumor CR rates during EBRT in younger patients (<50) with smaller tumors (100 cc) without nodal involvements as well as a positive correlation between PET FDG (18F-fluorodeoxyglucose)-SUV and CC primary tumor size.

## Introduction

Cervical cancer (CC) is the fourth most frequently diagnosed cancer in women with 570,000 new cases worldwide in 2018 [1]. Sequential external beam radiation therapy (EBRT) and brachytherapy (BT) with concurrent cisplatin remain the standard of care for locally advanced CC [2]. However, approximately one-third of CC patients in the United States who receive curative concurrent chemotherapy and radiation therapy (RT) eventually die from either local recurrence and/or metastasis with limited salvage options [3]. The incidence of CC-related deaths is much higher globally due to a lack of standardized screening programs and access to adequate care accounting for 311,000 cancer-related deaths in 2018 [1]. Salvage treatment options for CC recurrence include surgery, which can be morbid, and second- and third-line systemic treatments [3]. Further RT dose escalation to the primary tumor without grade 3-5 toxicities to the bowel, bladder, and rectum is challenging since the primary tumor receives a total biological equivalent dose of 2 Gy per fraction greater than 80 Gy when combined with EBRT and BT.

CC patients at high risk for recurrence and metastasis who otherwise could benefit from consolidative systemic treatments after curative chemo-RT are still being investigated [4,5]. An optimal time to decide on consolidative systemic treatments is near the end of EBRT. This is because CC primary tumor response (TR) on pre-brachytherapy imaging often varies from complete to minimal response between patients indicating significant biological heterogeneity. Various pathological and radiological factors have been identified as CC prognostic factors such as tumor size, TR, complete pathological response after chemo-RT, lymph node (LN) status, stage, age, RT duration, and maximum standardized uptake value (SUV) of 18F-fluorodeoxyglucose (FDG) avidity from positron-emission tomography (PET) scans [6-10]. However, histopathological and radiological factors associated with CC primary TR during EBRT as well as associations between such factors are minimally studied in CC. We performed an exploratory analysis seeking any associations between age, EBRT dose, tumor size, nodal involvement, or stage with complete response (CR) rates of the primary CC tumor assessed by MRI during EBRT. 

## Materials and methods

Study criteria

One hundred and twenty-four patients from January 2008 to July 2015 at Karmanos Cancer Institute, Wayne State University, and Detroit Medical Center with CC treated with concurrent chemotherapy (cisplatin) and RT consisting of EBRT and high-dose-rate brachytherapy (HDR-BT) were retrospectively screened for the study after approval from an institutional review board. The inclusion criteria for the study were a) pre-EBRT PET and magnetic resonance imaging (MRI) acquired for estimating initial tumor volume (GTV_INITIAL_); b) absence of non-regional metastatic disease; c) biopsy-proven cervical cancer; and d) completion of planned chemo-RT regimen. All patients were staged according to the 2009 International Federation of Gynecology and Obstetrics (FIGO) classification and pelvic and para-aortic nodal metastasis was considered as regional metastasis.

Radiation techniques

EBRT consisted of a whole pelvic RT of 45 Gy (1.8 Gy per fraction with 5 fractions per week). Gross LNs received a sequential EBRT boost. HDR-BT followed the whole pelvic EBRT. EBRT was delivered by either three-dimensional RT (3DRT) or intensity-modulated RT techniques.

Tumor and treatment measurements

EBRT planning was done on an Eclipse treatment planning system (Palo Alto, CA) using computed tomography (CT) scans obtained from a large-bore Somatom Sensation CT simulator (Siemens, Malvern, PA) after fusing with MRI and PET scans. GTV_INITIAL_ was estimated by contouring the primary tumor after fusing pre-EBRT MRI (MRI^1st^) and pre-EBRT PET scans with the planning CT scan (CT-SIM). A second MRI (MRI^2nd^) was obtained during the last two weeks of EBRT for HDR-BT planning and was used to estimate the final gross tumor volume (GTV_FINAL_) by contouring the primary tumor on MRI^2nd^. All tumor volumes were measured in cubic centimeters (cc). TR during EBRT was calculated by using the formula "TR = GTV_INITIAL_ - GTV_FINAL_." CR was defined as the absence of radiologically detectable primary tumor on MRI^2nd^. Partial radiological response (PR) was defined as 90% or less shrinkage but not CR of the primary tumor on MRI^2nd^. The SUV of the FDG avidity for the primary biopsy-proven tumor was obtained from respective PET scan reports. The durations between CT-SIM to first EBRT fraction (SIM-EBRT^1st^), CT-SIM to MRI^2nd^ (SIM-MRI^2nd^), and first EBRT fraction to MRI^2nd^ (EBRT^1st^-MRI^2nd^) were calculated from the respective dates. Follow-up was defined as the duration between the last day of either radiation or chemotherapy and the date of the last clinical evaluation without clinical or radiological evidence for any type of tumor recurrence. Time to recurrence was defined as the duration between the last day of either radiation or chemotherapy and the date that demonstrated either clinical or radiological evidence for any type of tumor recurrence. Recurrences (residual and or new lesions) within and outside the radiation field were labeled regional and non-regional recurrence, respectively. 

Statistical analysis

Spearman's rank correlation coefficient (rho) values were calculated to assess the relationships between age, tumor size, and SUV. Point estimates and 90% confidence interval (CI) estimates were calculated for each rho value. Fisher’s Z-transformation was used to enable the calculation of the 90% CI for rho. Point estimates and Wilson score-type CI estimates of CR rates were calculated. As exploratory analyses, CR rates were compared depending on the level of various demographic and clinical characteristics using Fisher’s exact test (two-sided). Point estimates and Wald-type 90% CI estimates of the odds ratio (OR) for CR rates were calculated by the level of those same patient characteristics. The 90% confidence level was chosen in view of the modest number of CR patients. For consistency, the 90% confidence level was also used for the CIs of the rho values as well. As additional exploratory analyses, the potential association of each patient characteristic with the odds of CR was assessed via univariable logistic regression (LR) models. No adjustments for multiple comparisons (i.e., test multiplicity) were made since all analyses of CR rates and ORs were exploratory. The goodness of fit of each LR model was assessed using the Hosmer and Lemeshow quantiles of risk method, and no evidence of lack of fit was observed. All analyses were performed using SAS version 9.4 statistical software. All OR estimates for the dichotomous exposure variables are imprecise (i.e., have rather wide, or sometimes very wide CIs) due to the small number of CR patients (n = 10), and are regarded as an exploratory analysis only.

## Results

Forty-eight patients met the inclusion criteria of the study. Seventy-nine percent of the patients were African American with a median age of 50 years (range: 22-85; Table 1). Stage IIB (40%) was the most common stage group followed by stages IB (33%), IIA (13%), IIIB (10%), and IIIA (4%) (Table 1). Eighty-eight percent of the patients had squamous cell carcinoma with 52% of the patients having nodal metastasis (Table 1). Six percent and 4% of the patients had adenocarcinoma and adenosquamous carcinoma, respectively, with one patient having cervical intra-epithelial neoplasia with micro-invasion (Table 1). Ninety-eight percent (47/48) of the patients received concurrent chemo-RT (Table 2). The Median EBRT dose to the pelvis was 45.0 Gy in 1.8 Gy per fraction (range: 45.0-52.2) (Table 2). Fifty-two percent of the patients received nodal boost radiation with doses ranging from 3.6 to 19.6 Gy in 1.8-2.0 Gy per fraction (Table 2). All patients completed HDR-BT in 4-5 fractions with dose per fraction ranging from 5.0 Gy to 7.5 Gy (Table 2). The durations of SIM-EBRT^1st^, SIM-MRI^2nd^, and EBRT^1st^-MRI^2nd^ were 8.0 days (range: 2.0-26), 41 days (range: 29-64), and 31 days (22-52), respectively (Table 2). Median GTV_INITIAL _and GTV_FINAL_ were 80 cc (range: 7.7-818) and 4.2 cc (range: 0.0-44), respectively (Table 2). Twenty-one percent (10/48) and 38% (18/48) of the patients had either a CR or PR at the time of MRI2^nd^, respectively. Median SUV from the PET scan was 15 (range: 4.3-28) (Table 2). The median EBRT dose at the time of the MRI^2nd^ was 37.8 Gy (range: 25-45 Gy). Fifteen percent and 17% of the patients had regional and distant recurrences, respectively after a median follow-up of 23.9 months (range: 0.5-79) (Table 2). Three patients were lost to follow-up. 

**Table 1:** Population characteristics (N = 48) N: number of patients; non-AA: non-African American; CIN: cervical intra-epithelial neoplasia. *Pelvic/para-aortic/retroperitoneal or inguinal nodes, diagnosed clinically or radiologically.

Parameter	No of patients
Age (years; median and range)	50 (22-85)
Race:	
African American	38 (79%)
Non-AA	10 (21%)
Stage:	
IB	16 (33%)
IIA	6 (13%)
IIB	19 (40%)
IIIA	2 (4%)
IIIB	5 (10%)
Pathology:	
Adenocarcinoma	3 (6%)
Adenosquamous carcinoma	2 (4%)
CIN with invasion	1 (2%)
Squamous cell carcinoma	42 (88%)
Regional nodal involvement*:	
No	23 (48%)
Yes	25 (52%)

**Table 2:** Treatment and tumor characteristics (N = 48) N: number of patients; EBRT: external beam radiation therapy; 3DCRT: three-dimensional conformal radiation therapy; IMRT: intensity-modulated radiation therapy; HDR: high-dose rate; BT: brachytherapy; SIM-EBRT^1st^: duration between CT-SIM to first EBRT fraction; SIM-MRI^2nd^: duration between CT-SIM to MRI^2nd^; EBRT^1st^-MRI^2nd^: duration between first EBRT fraction to MRI^2nd^; cc: cubic centimeters; GTV_INITIAL_: initial tumor volume; GTV_FINAL_: final tumor volume; SUV: maximum standardized uptake value of 18F-fluorodeoxyglucose (FDG) avidity from positron-emission tomography (PET). *Median.

Parameter	No of patients
EBRT dose to pelvis:	
45 Gy	47 (98%)
52.2 Gy	1 (2%)
Modality:	
3DCRT	32 (67%)
IMRT	16 (33%)
Nodal boost:	25 (52%)
HDR brachytherapy boost:	48 (100%)
Fractionation schemes:	
5.5 Gy × 5	18 (38%)
Alternate BT schemes	29 (60%)
Concurrent chemotherapy:	
Cisplatin	47 (98%)
None	1 (2%)
Time interval (days)*:	
SIM-EBRT^1st^	8.0 (2-26)
SIM-MRI^2nd^	41 (29-64)
EBRT^1st^-MRI^2nd^	31 (22-52)
GTV_INITIAL _(cc)	80 (7.7-818)
GTV_FINAL_ (cc)	4.2 (0.0-44)
SUV (maximum standardized uptake value)	15 (4.3-28)
Recurrence patterns:	
Regional	7 (15%)
Non-regional	8 (17%)
Regional and non-regional	5 (10%)
Time to recurrence (months)^*^	12 (1-34)
Follow-up (months)^*^	24 (0.5-79)

**Figure 1:**
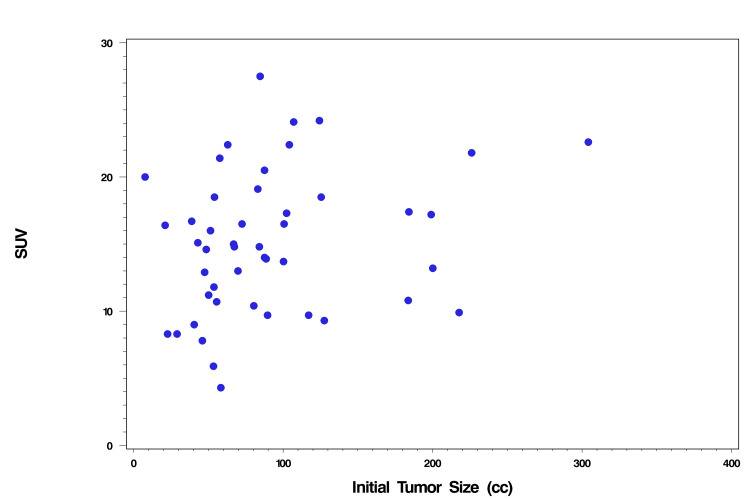
Scatter plot Scatter plot illustrating the relationship between SUV (from PET scan) and initial tumor size (GTV_INITIAL_; cc). One data point with GTV_INITIAL_ of 820 cc was excluded, as it was an extreme outlier. SUV: maximum standardized uptake value; PET: positron-emission tomography; GTV_INITIAL_: initial primary gross tumor volume.

A significant correlation (p = 0.03) was seen between SUV and GTV_INITIAL _with higher SUV associated with larger tumor size on Spearman's rank correlation analysis (Figure 1 and Table 3). No significant association was observed between SUV, age, and TR (Table 3). CR rates were higher for patients who were aged <50 years (29%) vs 13% for those aged ≥50 years (p = 0.2865), or with >37.5 Gy radiation dose (28%) vs 13% for dose ≤37.5 Gy at or before second MRI (p = 0.2917) (Table 4). Similarly, CR rates were higher for patients with GTV_INITIAL_ <100 cc (26%) vs 12% for GTV_INITIAL_ ≥100 cc (p = 0.4587), or with no nodes involved (30%) vs 12% with nodal involvement (p = 0.1615), or with stages IB or IIA (32%) vs 12% for stages IIB, IIIA, and IIIB (p = 0.1523) (Table 4). ORs for CR were calculated for continuous (age, GTV_INITIAL_, and radiation dose at the time of MRI^2nd^) and dichotomous variables (stage and nodal involvement) in Table 5. The OR for age was 0.962, hence the odds of a CR decreased by 3.8% with each additional year of age (Table 5). The odds of a CR for patients with a radiation dose of ≤37.5 Gy at or before the second MRI were 0.39 times that of patients who received a radiation dose >37.5 Gy (Table 5). For radiation dose at or before the second MRI as a continuously distributed exposure variable, the odds of a CR increased by approximately 18% for each additional 1.0 Gy of dose. The odds of a CR for patients with GTV_INITIAL_ <100 cc were 2.6 times that of the patients with GTV_INITIAL_ ≥100 cc (Table 5). The odds of a CR for patients with no nodes involved were 3.2 times that of the patients with nodes involved (Table 5). The odds of a CR for stage IB or IIA patients were 3.6 times that of stage IIB or IIIA or IIIB patients (Table 5).

**Table 3:** Spearman's rank correlation (Rho) statistics (N = 48) N: number of patients; CI: confidence interval; GTV_INITIAL_: initial tumor volume; GTV_FINAL_: final tumor volume; TR: tumor response; SUV: maximum standardized uptake value of 18F-fluorodeoxyglucose avidity from positron-emission tomography.

Variable	With variable	Sample correlation	90% CI	p-Value for H0: Rho = 0
SUV	Age	0.05	-0.19 to 0.29	0.73
SUV	GTV_INITIAL_	0.31	0.07 to 0.51	0.03
Age	GTV_INITIAL_	0.13	-0.12 to 0.36	0.39
TR	SUV	-0.07	-0.31 to 0.17	0.63
TR	Age	0.03	-0.21 to 0.27	0.83
TR	GTV_INITIAL_	-0.03	-0.27 to 0.21	0.83

**Table 4:** CR rates (N = 48) CR: complete response; N: number of patients; R: number of complete responders; CI: confidence interval; LL: lower limit; UL: upper limit; EBRT: external beam radiation therapy; GTV_INITIAL_: initial tumor volume; cc: cubic centimeters. Probability (p) value from Fisher’s exact test, two-sided.

Comparison variable	N	R	CR rate	Wilson type 90% CI (two-sided)		p-Value
				LL	UL	
Age (years):	48	10				0.2865
<50	24	7	0.292 (29.2%)	0.167	0.459	
≥50	24	3	0.125 (12.5%)	0.051	0.275	
EBRT dose at or before MRI^2nd^:	48	10				0.2917
≤37.5 Gy	23	3	0.130 (13.0%)	0.053	0.285	
>37.5 Gy	25	7	0.280 (28.0%)	0.160	0.443	
GTV_INITIAL_ (cc):	48	10				0.4587
<100	31	8	0.258 (25.8%)	0.152	0.403	
≥100	17	2	0.118 (11.8%)	0.040	0.301	
Nodes involved:	48	10				0.1615
No	23	7	0.304 (30.4%)	0.174	0.476	
Yes	25	3	0.120 (12.0%)	0.049	0.265	
Stage:	48	10				0.1523
IB or IIA	22	7	0.318 (31.8%)	0.183	0.494	
IIB or IIIA or IIIB	26	3	0.115 (11.5%)	0.047	0.256	

**Table 5:** Odds ratios for complete response (N = 48) N: number of patients; R: number of complete responders; ln (GTV_INITIAL_): natural logarithmic transformation of initial gross tumor volume measured from CT simulation; CI: confidence interval; LL: lower limit; UL: upper limit; EBRT: external beam radiation therapy; GTV_INITIAL_: initial tumor volume; cc: cubic centimeters. Probability (p) value from Wald test, two-sided.

Comparison variable	N	R	Odds ratio	Wald type 90% CI (two-sided)		p-Value
				LL	UL	
Age (years):	48	10	0.962	0.909	1.018	0.2570
<50	24	7	2.882	0.821	10.116	0.1655
≥50	24	3	1.000			
EBRT dose at or before MRI^2nd^:	48	10	1.178	1.016	1.365	0.0681
≤37.5 Gy	23	3	0.386	0.110	1.352	0.2117
>37.5 Gy	25	7	1.000			
ln (GTV_INITIAL_; cc):	48	10	0.355	0.117	1.081	0.0682
<100	31	8	2.609	0.486	14.004	0.2634
≥100	17	2	1.000			
Nodes involved:						
No	23	7	3.208	0.717	14.350	0.1272
Yes	25	3	1.000			
Stage:						
IB or IIA	22	7	3.578	0.798	16.047	0.0960
IIB or IIIA or IIIB	26	3	1.000			

## Discussion

The major finding from the study is the positive correlation between FDG-SUV and GTV_INITIAL_ in CC. FDG-SUV is an indirect measure of glucose metabolism within a specific anatomical region of interest, which in turn is a measure of metabolic activity [11]. Upregulated metabolic activity compared to physiological levels is a surrogate marker for malignancy [11]. Elevated FDG-SUV above the physiological threshold is correlated with inferior cancer control outcomes such as poorer local control and higher metastatic rates in cancers of the lung, esophagus, and cervix [12,13]. However, limited data exist to examine correlations between tumor characteristics and patient demographics with FDG-SUV in CC [14,15]. The positive correlation between CC size and FDG-SUV and larger CR rates for tumors <100 cc compared to tumors >100 cc indicates that larger CC tumors are biologically more aggressive. There could be a variety of reasons underlying these observations. First, larger tumors have more hypoxic and necrotic niches, which create zones of radiation resistance within the tumor [16]. Secondly, an increase in tumor volume with elevated FDG-SUV indicates rapidly dividing tumor cells and thus a higher likelihood of acquiring treatment-resistant mutations [16]. The strength of this observation is further highlighted in the current study by our method of estimating CR rates and TR using tumor volumes drawn on a CT-SIM after being registered with PET and MRI. This is because tumor volumes are better prognostic markers compared to tumor dimensions [9,10,17] and the radiological accuracy of MRI in soft tissue delineation is well established [18-20].

Age is a well-established independent prognostic factor for gynecological malignancies with advanced age associated with poorer survival [21,22]. CR from EBRT was higher in women younger than 50 years old in the current study. The biological mechanisms underlying this phenomenon in CC are unknown with various mechanisms that have been proposed in recent years. Chronic persistent inflammation by HPV, the etiologic agent underlying CC, is vital to the evolution of CC-infected cervical stroma to invasive cancer [21,22]. Several studies have shown HPV replication suppressive mechanisms by estrogen [21,22]. Physiological levels of estrogen decrease with age especially after menopause, and thus, the virulence and consequent tumorigenic propensity of HPV might be higher in elderly women, creating biologically radio-resistant tumors [21,22]. Advanced age is also associated with a weaker immune system and thus a higher likelihood of immune evasion by the CC tumor cells from tumor-infiltrating lymphocytes such as cytotoxic T cells and macrophages [23]. Lower TR rate to EBRT with advancement in age in our study could thus be the consequence of a multitude of such reasons.

The current study was done using the 2009 FIGO staging system and showed that the patients with stage IB or IIA had numerically higher CR rates. This is in accordance with current literature that has shown significant differences in five-year progression-free survival between 2009 and 2018 FIGO staging [6,7]. Stage and nodal metastasis are independent prognostic factors in CC [3,4,6,24,25]. One of the major differences between the 2009 and the 2018 staging systems is the establishment of stage IIIC that groups CC patients with LN metastasis [6,7,26]. In the 2009 staging, LN involvement was only used for staging if clinically apparent [6,7,26]. Fifty-two percent of our patients had LN involvement and thus the absence of any statistically significant association between CR rate and the stage could be attributed to the presence of LN involvement in many of our stage II and stage III patients who could have been categorized as a separate group. This is further illustrated by our observation that patients with nodal involvement had a poorer CR rate, and by odds of CR 3.2 higher in patients without nodes. Recent studies have demonstrated improved survival in patients who received prophylactic para-aortic EBRT with positive pelvic nodes as well as poorer three-year distant metastasis-free survival in patients with matted or nodes greater than 1.5 cm [4,25]. Biologically, nodal metastasis indicates that the respective primary tumor has acquired the molecular properties for regional spread through lymphatics [24,25,27]. Such tumors are more radio-resistant and could explain the higher CR rates seen in our patients without nodal metastasis [24,25,27]. In the current study, 15% of patients also had a regional recurrence and 17% had a non-regional recurrence. A potential explanation for the high recurrence pattern could be that 52% and 54% of our patients had nodal metastasis and were locally advanced (IIB/IIIA/IIIB) CC, respectively [4,6]. A relationship between CR rates and post-treatment recurrence patterns was not pursued due to the retrospective nature of the study. 

The major limitation of the study is due to the retrospective nature of the study and consequently, the logistical variations in the dose delivered and the time interval between the GTV_INITIAL_ and GTV_FINAL_, which are critical in accurately estimating CR and TR [28,29]. TR and CR estimations are dependent on the dose at the time of the MRI^2nd^ and the duration between CT-SIM and MRI^2nd^ since the largest radiation dose given in the shortest interval leads to the greatest TR [29,30]. This is in accordance with several prior studies that demonstrated better outcomes in CC if the entire course of RT with concurrent chemotherapy is completed within 56 days [20,28-30]. The EBRT dose at the time of the MRI^2nd^ in our study varied with a large dose range (25-45 Gy) with a median dose of 37.8 Gy and thus patients who received a larger dose more often had a greater TR. This is further supported by the Fisher’s exact test we performed that showed a higher clinical likelihood of CR on the day of MRI^2nd^ if the patient received greater than 37.5 Gy. The median EBRT^1st^-MRI^2nd^ duration was 31 days with a large range (22-52 days) and thus patients who had MRI^2nd^ showing better TR most likely received an EBRT dose >37.5 Gy. Similarly, the median SIM-EBRT^1st^ duration, which was 8.0 days with a large range (2-26 days), also could have contributed to the under-estimation of TR in the study since delays in the initiation of EBRT can lead to an increase in the size of the tumor.

## Conclusions

The current study identified a positive correlation between cervical cancer primary tumor volume and FDG-SUV and CR rates of the primary tumor during EBRT were higher for patients who had any of these five characteristics: age <50, or >37.5 Gy radiation dose at or before the second MRI, or primary tumor size <100 cc, or no nodes involved, or stage IB or IIA. Further prospective studies are necessary to validate the findings and to delineate the histopathological and demographic factors that can predict CC TR during EBRT as well as to study whether TR during EBRT has prognostic significance. Such studies could set the stage for future trials that identify high-risk CC patients who might benefit from consolidated systemic treatments. 
